# Thickness-confined metastable phase transitions drive large piezoelectricity in ultrathin BiFeO_3_

**DOI:** 10.1126/sciadv.aeb7174

**Published:** 2026-03-13

**Authors:** Shuang-Jie Chen, Meixiong Zhu, Jing-Hui Wang, Tongtong Shi, Jiaqi Liu, Yujia Wang, Yinlian Zhu, Xiu-Liang Ma, Zuhuang Chen, Yunlong Tang

**Affiliations:** ^1^ Shenyang National Laboratory for Materials Science, Institute of Metal Research, Chinese Academy of Sciences, Wenhua Road 72, Shenyang 110016, China.; ^2^ School of Materials Science and Engineering, University of Science and Technology of China, Wenhua Road 72, Shenyang 110016, China.; ^3^ Bay Area Center for Electron Microscopy, Songshan Lake Materials Laboratory, Dongguan 523808, China.; ^4^ School of Materials Science and Engineering, Hunan University of Science and Technology, Xiangtan 411201, China.; ^5^ Quantum Science Center of Guangdong-HongKong-Macau Greater Bay Area, Shenzhen 518045, China.; ^6^ Institute of Physics, Chinese Academy of Sciences, Beijing 100190, China.; ^7^ State Key Laboratory of Precision Welding and Joining of Materials and Structures, School of Materials Science and Engineering, Harbin Institute of Technology, Shenzhen 518055, China.

## Abstract

Pursuing high-performance lead-free piezoelectrics beyond classical thickness limits remains challenging. This study identifies a transitional phase between rhombohedral and tetragonal structures in strained ultrathin BiFeO_3_ layers within (BiFeO_3_/Ca_0.96_Ce_0.04_MnO_3_)_4_ multilayer films grown on LaAlO_3_ substrates. Atom-scale studies and quantitative electromechanical atomic force microscopy revealed that the transitional phase facilitates continuous polarization rotation in ultrathin BiFeO_3_ layers. This effect enhances the piezoelectric responses of the multilayer films and yields a giant piezoelectric coefficient (*d*_33_ ≈ 30 picometers per volt) for films containing 16–unit cell BiFeO_3_ layers, which is over four times higher than conventional rhombohedral BiFeO_3_. Phase-field simulations confirmed a thickness-dependent electromechanical coupling regularity, behaving as the coexistence of transitional/tetragonal mixed phases and dense nanodomains in strained ultrathin BiFeO_3_ layers. This work breaks the thickness limit of single-layer BiFeO_3_ for electromechanical applications and proposes a thickness-domain design strategy for lead-free piezoelectric heterostructures.

## Teaser

A metastable transitional phase in ultrathin BiFeO_3_ layers yields large piezoelectric responses in multilayer films.

## INTRODUCTION

Piezoelectric materials are enabling technologies for energy conversion and sensing in electronics, yet their widespread adoption is hindered by the toxicity of lead-based systems [e.g., Pb(Zr*_*x*_*Ti_1−*x*_)O_3_, (1 − *x*)(Pb(Mg_1/3_Nb_2/3_)O_3_) − *x*(PbTiO_3_)] (*1*–*3*). BiFeO_3_ has emerged as a leading lead-free alternative due to its robust multiferroicity and room temperature (RT) functionality (*4*, *5*). With a complex crystal structure and diverse physical properties (*6*), BiFeO_3_ offers deep insights into multiferroic coupling mechanisms under various physical conditions (*7*–*11*). For example, its unique properties endow BiFeO_3_ with great potential in integrated piezoelectric micro-electromechanical systems (MEMS) devices (*12*), enhancing piezoelectric catalyst performance (*13*), and in flexible piezoelectric materials (*14*), enabling efficient energy conversion and technological innovation. However, the “thickness effect” severely restricts BiFeO_3_ application in thin film miniaturized devices. Below 30 nm, interfacial relaxation and phase instability cause a marked decline in electromechanical coupling, limiting its thin film performance (*15*).

A fundamental challenge in the field is the lack of a universal design principle to decouple piezoelectric performance from film thickness (*9*, *15*). In single-layer systems, due to interfacial lattice mismatch and the critical thickness effect, ultrathin BiFeO_3_ films (<30 nm) experience substrate-induced structural confinement, which suppresses the formation of morphotropic phase boundaries (*15*) and stabilizes single-phase configurations. Conversely, thick films (>50 nm) are susceptible to strain relaxation via dislocation nucleation or secondary phase precipitation, introducing domain wall (DW) pinning and deteriorating piezoelectric performance (*5*, *9*, *16*–*18*).

A practical solution is imperative for overcoming these formidable challenges. Here, we introduce a multilayer heterostructure approach that leverages cumulative interfacial strain to stabilize metastable phases in ultrathin BiFeO_3_ films. By systematically tuning single-layer BiFeO_3_ thickness [20-8 unit cells (U.C.)] in (BiFeO_3_/Ca_0.96_Ce_0.04_MnO_3_)_4_ multilayers, we identify a strain-driven symmetry-lowered transitional phase with enhanced electromechanical coupling, overcoming the thickness limitation and establishing a scalable pathway for lead-free piezoelectric devices. Specifically, through thickness engineering (20-8 U.C.), we systematically induced phase evolution from periodic trapezoid rhombohedral/tetragonal mixed phases to tetragonal/transitional mixed phases and then transitional/rhombohedral mixed phases, as confirmed by transmission electron microscopy. Notably, the 16 U.C. BiFeO_3_ layer exhibits optimal piezoelectric performance, characterized by continuous polarization rotation, increased domain wall density, and a piezoelectric coefficient (*d*_33_) four times higher than conventional rhombohedral phase (R-phase) films through quantitative electromechanical atomic force microscopy using an interferometric displacement sensor (IDS) analysis and phase simulated analysis.

Beyond BiFeO_3_, the multilayer heterostructure strategy offers a universal platform for overcoming thickness limitations in functional oxides, including ferroelectrics, multiferroics, and thermoelectric (*7*). For MEMS and flexible electronics (*19*), this approach enables the miniaturization of piezoelectric devices without performance degradation, aligning with global efforts to develop sustainable, lead-free technologies (*20*, *21*). Future research should focus on integrating these heterostructures with scalable fabrication techniques and exploring their application in energy harvesting and adaptive sensing systems (*22*).

## RESULTS

### Characterization of macroscopic growth quality of BFO multilayer film

A series of BiFeO_3_/Ca_0.96_Ce_0.04_MnO_3_ (BFO/CCMO) films with varying thicknesses on LaAlO_3_ (001) (LAO) substrates was prepared by pulsed laser deposition. The thickness of each BFO layer in different samples is 20 U.C., 16 U.C., and 8 U.C., respectively, while each CCMO layer is approximately 13 U.C. thick. BFO is an RT multiferroic material with coupled ferroelectric and antiferromagnetic properties. At RT, BFO exhibits a rhombohedral structure with pseudocubic lattice parameter of 3.965 Å (*5*, *23*). CCMO, a doped conductive oxide used as an electrode in epitaxial perovskite oxide film systems, has a lattice parameter of 3.77 Å (*24*). The in-plane lattice mismatch between LAO substrate and the BFO film is 4.66%.

As shown in figs. S1 and S2, x-ray diffraction (XRD) of (BFO/CCMO)_4_ multilayers with varying thicknesses reveals a systematic leftward shift of diffraction peaks with increasing thickness. Compared to single-layer BFO thin films grown on SrTiO_3_ (STO) substrates, the out-of-plane lattice parameters show greater tetragonality in the 20 U.C. and 16 U.C. samples. This may indicate a tetragonal-like phase in these BFO films. Unlike the strain release behavior in single-layer BFO, the 20 U.C. and 16 U.C. samples exhibit three cleavage peaks. High-resolution XRD reciprocal space mapping (RSM) data showed that the diffraction spot exhibits diffuse scattering in the 20 U.C. BFO sample, which becomes more obvious at the 16 U.C. sample. When the thickness decreases to 8 U.C., the diffuse spots show relatively scattering intensity with clear oscillating fringes. To figure out the microstructural origins of this behavior, we used high-angle annular dark-field scanning transmission electron microscopy (HAADF-STEM) imaging (*25*) and geometric phase analysis (GPA) (*26*).

### The domain structure of BFO multilayer evolved with thickness

**Fig. 1.**
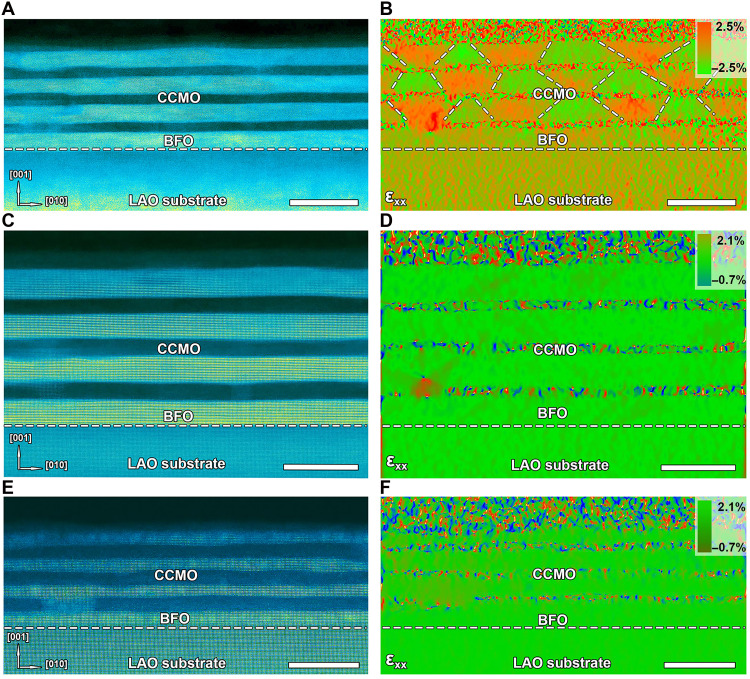
Low-magnification HAADF-STEM images and corresponding GPA analysis of LAO/(BFO/CCMO)_4_ films with different thicknesses. (**A**) HAADF-STEM image of LAO/(BFO/CCMO)_4_ with 20 U.C. monolayer BFO. (**B**) In-plane strain based on GPA analysis corresponding to (A). (**C**) HAADF-STEM image of LAO/(BFO/CCMO)_4_ with 16 U.C. monolayer BFO. (**D**) In-plane strain based on GPA analysis corresponding to (B). (**E**) HAADF-STEM image of LAO/(BFO/CCMO)_4_ with 8 U.C. monolayer BFO. (**F**) In-plane strain based on GPA analysis corresponding to (E). Scale bars, 30 nm [(A) and (B)] and 20 nm [(C), (D), (E) and (F)].

HAADF-STEM imaging was used to characterize the low-magnification morphology of this series of films, accompanied by quantitative GPA analysis. The corresponding results are shown in Fig. 1 and fig. S3. Figure 1 (A, C, and E) presents low-magnification HAADF-STEM images of samples with single-layer BFO thicknesses of 20 U.C., 16 U.C., and 8 U.C., respectively. Subtle interface undulations could be observed in these BFO/CCMO multilayers, particularly for the 20 U.C. BFO layers, suggesting a thickness-dependent evolution of microstructural features.

Figure 1A shows a HAADF-STEM image of the 20 U.C. BFO sample, with corresponding in-plane and out-of-plane strains extracted via GPA (Fig. 1B and fig. S3, A and B). The BFO film exhibits a unique periodic strain distribution featuring trapezoidal domains, possibly suggesting some unique domain configurations. In the two-dimensional (2D) in-plane strain map, the red trapezoidal regions exhibit an in-plane strain of +2.5%, corresponding to an average out-of-plane strain of ~8%, while the green trapezoidal regions show an in-plane compressive strain of −2.5% with a notable out-of-plane strain of ~18%. On the basis of literature correlations between strain relaxation and structural evolution in compressed BFO films, this periodic domain structure may originate from alternating R-phase and tetragonal phase (T-phase).

Notably, as film thickness decreases to 16 U.C., this periodic R/T phase gradually disappears, replaced by a subtle increase in local in-plane strain with minimal out-of-plane strain variation (maintaining ~17 ± 2% fluctuation), as shown in Fig. 1 (C and D) and fig. S3 (C and D). In the 8 U.C. BFO sample, the in-plane strain distribution becomes homogeneous, while the out-of-plane strain fluctuates between 8 and 12% (Fig. 1, E and F, and fig. S3, E and F) In addition, energy-dispersive spectroscopy mapping reveals distinct spatial contrast for the specific elements Bi, Fe, Ca, Ce, Mn, Al, and La, indicating high-quality chemical homogeneity in the BFO multilayer system (fig. S4)

**Fig. 2.**
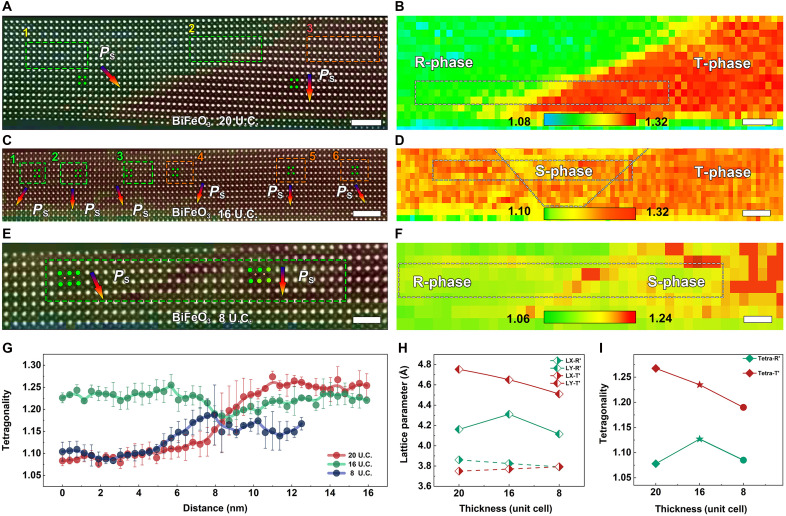
Polarization and tetragonal analysis of (BFO/CCMO)_4_ thin films with varying thicknesses. (**A**) Atomic-scale HAADF-STEM image of 20 U.C. BFO layer. (**B**) 2D tetragonality distribution in 20 U.C. BFO layer based on peak-finding fitting results. (**C**) Atomic-scale HAADF-STEM image of 16 U.C. BFO layer. (**D**) 2D tetragonality distribution in 16 U.C. BFO layer based on peak-finding fitting results. (**E**) Atomic-scale HAADF-STEM image of 8 U.C. BFO layer. (**F**) 2D tetragonality distribution in 8 U.C. BFO layer based on peak-finding fitting results. (**G**) Statistical distribution of tetragonality values corresponding to (B), (D), and (F). (**H**) Lattice parameters of Rʹ-like and Tʹ-like phases with varying thickness. (**I**) Statistical tetragonality of Rʹ-like and Tʹ-like phases versus film thickness. Scale bars, 2 nm [(A) and (B)], 2.5 nm [(C) and (D)], and 1 nm [(E) and (F)].

**Fig. 3.**
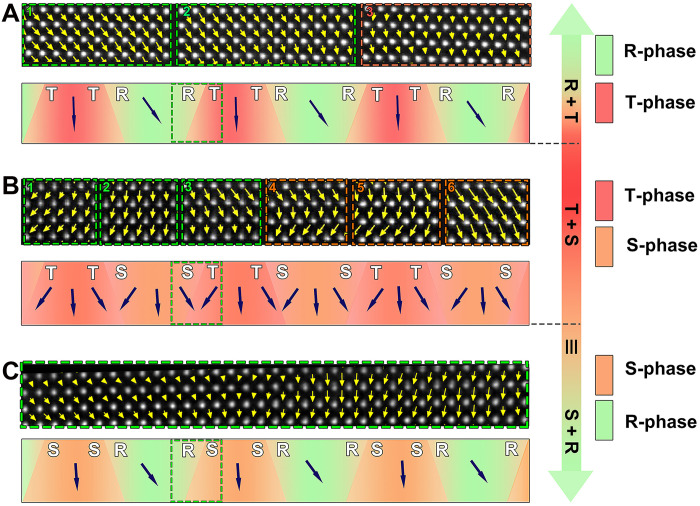
Polarization analysis and schematic diagram based on peak fitting in BFO films with different thicknesses. (**A**) 2D polarization vector map corresponding to the boxed area in Fig. 2A, alongside a schematic diagram of the polarization in the 20 U.C. BFO layer. (**B**) 2D polarization vector map corresponding to the boxed area in Fig. 2C with a corresponding schematic of the polarization in the 16 U.C. BFO layer. (**C**) 2D polarization vector map corresponding to the boxed area in Fig. 2E, accompanied by a schematic of the polarization in the 8 U.C. thick BFO layer.

Further analyses of the polarization structure and tetragonality of the BFO layers in (BFO/CCMO)_4_ multilayers are presented in Figs. 2 and 3. Figure 2 (A, C, and E) shows atomic-scale HAADF-STEM images of 20 U.C., 16 U.C., and 8 U.C. thick BFO samples, with corresponding tetragonality maps in Fig. 2 (B, D, and F). Analysis reveals distinct polarization orientations in colored regions of the BFO layers: The green rectangular area in Fig. 2A exhibits polarization inclined diagonally downward, whereas the red overlay region shows downward polarization. The tetragonality values in these regions (Fig. 2B) indicate a minimum of ~1.08 (approaching the R-phase BFO reported in literature) (*9*) in green areas and ~1.32 in red areas, consistent with the T-phase BFO structure. Notably, when the BFO thickness is reduced to 16 U.C., the tetragonality not only decreases to 1.13 to 1.25, but also the polarization exhibits a slight rotation in varying directions. Further thinning to 8 U.C., the tetragonality fluctuates between 1.08 and 1.20, while the polarization direction closely resembles behavior observed at R/T domain walls, as shown in Fig. 2 (D and F), respectively. Figure 2G presents a statistical analysis of tetragonality in the white dashed rectangular regions in Fig. 2 (B, D, and F). At 20 U.C. BFO sample, the tetragonality exhibits a continuous transition from 1.08 to 1.25, consistent with the R/T phase boundary behavior. For the 16 U.C. BFO sample, tetragonality variations narrow to the range of 1.24 to 1.21, reflecting thickness-dependent structural evolution under epitaxial strain. At 8 U.C. BFO sample, tetragonality follows a similar tendency to that at the R/T phase boundary, but the values fluctuate between 1.08 and 1.16. We define the S-phase as a strained transitional regime with a tetragonality (c/a ratio) of 1.08 to 1.20. It is not a thermodynamic phase, but a metastable state composed of monoclinic variants that exhibit a nominal tetragonality while maintaining monoclinic symmetry. Under changing strain conditions, strain is released via polarization rotation, which drives transitions between the monoclinic variants. This process concurrently alters the lattice parameters, manifesting as the pronounced changes in tetragonality that characterize the S-phase. (*23*). We attribute this to the formation of a mixed R/S phase structure, distinct from single-layer BFO films grown on LAO substrates (*9*, *15*).

Therefore, to statistically analyze variations in tetragonality and lattice parameters across phase structures at varying thicknesses, we define the phase with larger tetragonality in samples as the Tʹ phase and the phase structure with relatively smaller tetragonality as the Rʹ phase for easy distinction. The variations in their lattice parameters and tetragonality are shown in Fig. 2 (H and I), respectively. For the 20 U.C. sample, the out-of-plane lattice parameter of the Rʹ phase is 4.16 Å, and the in-plane lattice parameter is 3.86 Å. The out-of-plane lattice parameter of the Tʹ phase is 4.75 Å, and the in-plane lattice parameter is 3.75 Å. The corresponding tetragonalities are 1.078 and 1.268, respectively; for the 16 U.C. sample, the out-of-plane lattice parameter of the Rʹ phase is 4.31 Å, and the in-plane lattice parameter is 3.83 Å. The out-of-plane lattice parameter of the Tʹ phase is 4.65 Å, and the in-plane lattice parameter is 3.77 Å. The corresponding tetragonalities are 1.127 and 1.235, respectively; for the 8 U.C. sample, the out-of-plane lattice parameter of the Rʹ phase is 4.12 Å, and the in-plane lattice parameter is 3.79 Å. The out-of-plane lattice parameter of the Tʹ phase is 4.51 Å, and the in-plane lattice parameter is 3.79 Å. The corresponding tetragonalities are 1.085 and 1.19. The above statistical results in different samples are basically consistent with the fluctuation range in Fig. 2G.

Meanwhile, 2D peak-finding Gaussian fitting analysis (*27*) was also performed on the polarization distributions. As shown in Fig. 3A, the polarization direction in the 20 U.C. sample rotates slightly at domain walls while approaching the common R-phase and T-phase distributions away from the walls. For the T/S mixed phases present in the 16 U.C. sample (Fig. 3B), polarization vectors rotate and diverge within the BFO layer. In the 8 U.C. sample (Fig. 3C), the polarization vectors gradually approach the distributions in the R-phase and S-phase. Therefore, on the basis of Figs. 2 and 3, we could conclude that the microstructural evolution in BFO multilayers differs from that in monolayer BFO film. In monolayer BFO films below 30-nm thickness, interfacial confinement effects suppress tetragonality within a few unit cells near the substrate interface. Beyond this constrained region, a homogeneous T-like phase emerges to achieve strain relaxation (*9*). By contrast, our multilayers exhibit a mixed R/T phase–T/S phase–S/R phase transition with decreasing thickness.

### Characterization of piezoelectric properties of BFO multilayer films

**Fig. 4.**
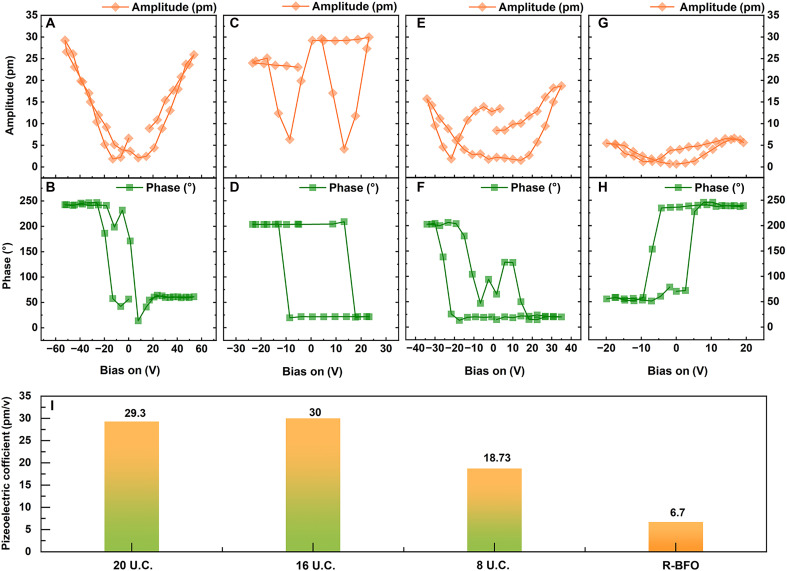
Thickness-dependent piezoelectric response and *d*_33_ statistics. (**A** and **B**) IDS-coupled amplitude and phase hysteresis loops of LAO/(BFO/CCMO)_4_ sample with 20 U.C. monolayer BFO. (**C** and **D**) IDS-coupled amplitude and phase hysteresis loops of LAO/(BFO/CCMO)_4_ sample with 16 U.C. monolayer BFO. (**E** and **F**) IDS-coupled amplitude and phase hysteresis loops of LAO/(BFO/CCMO)_4_ sample with 8 U.C. monolayer BFO. (**G** and **H**) IDS-coupled amplitudes and phase hysteresis loops of R-BFO sample with a thickness of 40 nm. (**I**) Statistical piezoelectric coefficients of different BFO multilayer systems (20 U.C., 16 U.C., and 8 U.C.) and STO/BFO system, respectively.

To investigate the influence of phase structure changes in BFO multilayers on their piezoelectric properties, we combined IDS (*28*) and piezoelectric force microscopy (PFM) (IDS-PFM) technology. The IDS-PFM technology could quantitatively measure the piezoelectric response of the BFO multilayer system, which could reduce the effect of electrostatic energy. Corresponding results are shown on Fig. 4. Figure 4 (A and B) presents the local PFM amplitude and phase hysteresis loops of the LAO/(BFO/CCMO)_4_ sample with a BFO thickness of 20 U.C., with a maximum out-of-plane amplitude of 29.3 pm; Fig. 4 (C and D) corresponds to the PFM amplitude and phase hysteresis loops of the 16 U.C. sample, with a maximum out-of-plane amplitude of 30 pm; Fig. 4 (E and F) shows the test results of the 8 U.C. sample, with a maximum out-of-plane amplitude of 18.73 pm; Fig. 4 (G and H) displays the local PFM amplitude and phase hysteresis loops of the sample with a single-layer R-phase BFO thickness of 40 nm (fig. S5). The statistical results of their piezoelectric coefficients are shown in Fig. 4I. The measured *d*_33_ uncertainties for these multilayer films range from ~± 2.3 pm/V for the 16 U.C. sample to less than ±0.5 pm/V for the R-BFO sample.

According to the statistical results from Fig. 4I, piezoelectric coefficients of the BFO multilayer film system are notably enhanced compared to those of the R-phase BFO. IDS-PFM measurements of the out-of-plane *d*_33_ coefficient in ferroelectric films indicate thickness-dependent piezoelectric response. Theoretically, piezoelectric responses scale positively with film thickness, resulting in higher output from thicker films than thinner ones (*29*). On the basis of the above results, the piezoelectric coefficient of the 16 U.C. BFO sample is slightly higher than that of 20 U.C. BFO sample and approximately 4.47 times higher than that of R-phase BFO, despite the theoretical expectation that reduced thickness lowers piezoelectric response. Even at 8 U.C. BFO sample, the piezoelectric coefficient remains 2.79 times enhancement over single-layer R-phase BFO.

**Fig. 5.**
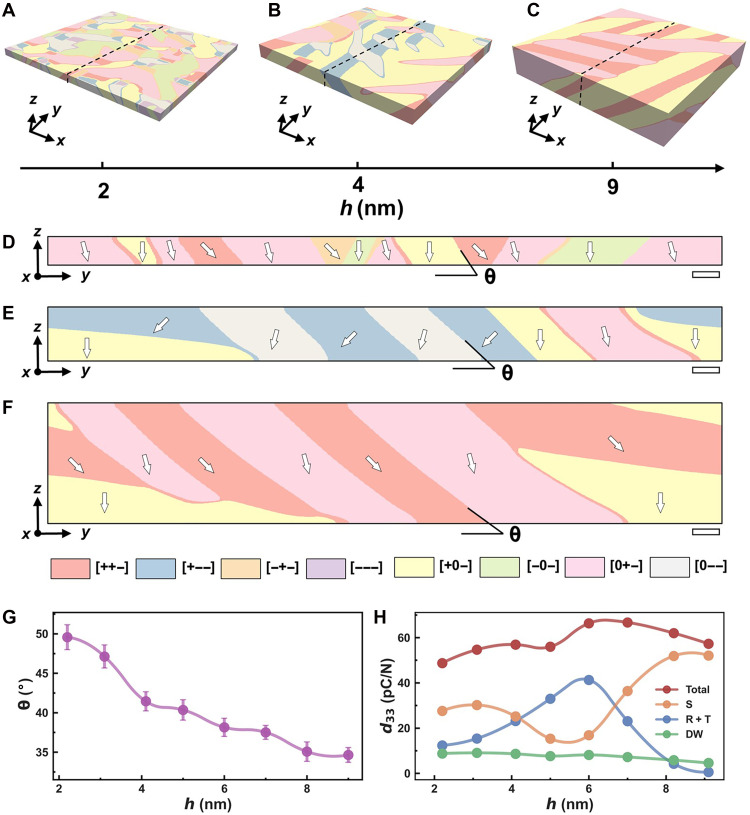
Phase-field simulations of thickness-dependent domain morphology and piezoelectric response in BFO thin films. (**A** to **C**) 3D domain configurations of BFO films with thicknesses of 2, 4, and 9 nm, respectively. (**D** to **F**) Cross-sectional domain maps extracted from slices normal to the [100] crystallographic axis, as indicated by black dashed lines in (A) to (C). Distinct ferroelastic variants are color-coded. (**G**) Variation of the angle θ between the polarization vectors of different ferroelastic variants and the film/substrate interface versus BFO thickness. (**H**) Thickness dependence of the out-of-plane piezoelectric coefficient *d*_33_ derived from the simulations. Scale bars, 5 nm [(D) to (F)].

To clarify the thickness-dependent piezoelectric response evolution observed experimentally, we performed phase-field simulations to investigate the domain structure morphology and piezoelectric properties of BFO films grown on LAO substrates. As shown in Fig. 5 (A to C), the domain size increases notably with film thickness increase, while the domain wall density decreases markedly from 0.42 nm^−1^ at 2-nm thickness to 0.18 nm^−1^ at 9-nm thickness (fig. S6).

The cross-sectional polarization distributions in Fig. 5 (D to F) indicate that the domain walls gradually tilt toward the film/substrate interface and become sharper and clearer as the film thickness increases. Statistical analysis of domain wall tilt angles (θ) at different thicknesses (Fig. 5G) demonstrates a gradual decrease with increasing thickness, stabilizing at 9 nm. We further evaluated the variation of the effective piezoelectric coefficient of BFO films with thickness, as shown in Fig. 5H. This coefficient exhibits a nonmonotonic dependence on thickness, increasing initially and then decreasing as thickness further increases. To elucidate the microscopic origin of the enhanced piezoelectric response, we performed a phase-resolved decomposition of the simulated response into contributions from the S, R, T, and DW. As shown in Fig. 5H and fig. S7, the analysis reveals that the enhancement is dominated by the emergence and progressive stabilization of the S-phase, which accounts for nearly 60% of the total piezoelectric response in the optimal thickness regime. While a reduction in thickness leads to an increased DW density, the resulting contribution remains minor and does not play a dominant role in the piezoelectric enhancement, given that DWs occupy only a limited portion of the volume.

In addition, we also note that the reduced polarization rotation barriers within the S-phase and at the T/S boundary, as quantified in fig. S8 by our solid-state nudged elastic band (SSNEB) calculations (*30*), provide direct microscopic support for the proposed mechanism. Figure S8 (A and B) shows that the monoclinic S-phase mediates a continuous polarization rotation pathway linking the R and T states, characterized by low curvature in polarization space and a smooth evolution of the rotation angle near the T/S region. As shown in fig. S8C, the direct transformation between the R and T minima requires overcoming a single barrier of 164 meV/formula unit (f.u.) By contrast, when the rotation proceeds via the intermediate S-phase, the process follows a two-step pathway, with an R-to-S barrier of ~104 meV/f.u. and a substantially smaller S-to-T barrier of about 57 meV/f.u. Although the cumulative barrier along this indirect pathway (161 meV/f.u.) is slightly lower than that of the direct R-T transition, the introduction of the S-phase effectively partitions the rotation barrier into two smaller steps, resulting in a flattened angular energy landscape and facilitating a smoother polarization rotation in the vicinity of the T/S region.

We suggest that the enhancement of piezoelectric response in the BFO system may originate from the mixed phase boundary or the polarization rotation in the strained intermediate phase of BFO. Experimental results confirm that stabilization of a distorted intermediate phase more effectively generates high piezoelectric response through polarization rotation, compared to the enhancement mechanism induced by the R/T phase boundary. On the one hand, the critical S-phase not only has an adjustable tetragonal distortion in the range of 1.20 to 1.08 but also allows easier rotation of its polarization direction. On the other hand, theoretical calculations with the phase-field simulation show that the increase of domain wall density in T/S mixed sample also contributes to the improvement of piezoelectric performance.

## DISCUSSION

Piezoelectric thin films, particularly lead-free alternatives, have garnered much attention for their potential in next-generation microelectronics and electromechanical devices (*20*, *31*). Among them, BFO systems exhibit exceptional promise due to their tunable functional properties under strain engineering (*5*, *7*, *11*, *23*, *32*). In BFO-based systems, the piezoelectric performance of BFO is typically modulated through the following approaches: (i) inducing phase transitions at the morphotropic phase boundary via misfit strain engineering to enhance piezoelectric response (*9*); (ii) leveraging strain anisotropy to stabilize high-performance monoclinic phases (*16*); and (iii) using chemical doping to achieve multiphase coexistence (*33*–*35*). However, these strategies remain confined to single-layer architectures and thickness limitations. Critically, deviations from optimal thickness, whether excessive or insufficient, could compromise domain stability and degrade electromechanical properties, ultimately limiting practical applications. Therefore, it is crucial to propose a thickness optimization strategy to avoid this phenomenon.

Here, we elucidate the thickness-dependent phase transitions and piezoelectric enhancement in BFO multilayers through an integrated approach combining atomic-scale characterization and phase-field simulations. This study reveals the structural evolution of BFO multilayer films under huge compressive strain and demonstrates their enhanced piezoelectric response compared to R-BFO thin films. Through a series of HAADF-STEM images and strain analysis of BFO films with varying thicknesses, we observed that the evolution of domain structures in BFO is strongly dependent on film thickness. Particularly in 16 U.C. BFO samples, a mixed T-like phase and S-phase were observed, resulting in an easier rotation of the tetragonal lattice, which could contribute to the high piezoelectric performance of BFO. When the thickness of the film increases to 20 U.C., it becomes a periodic trapezoidal R/T phase structure. When the thickness of the film is reduced to 8 U.C., the phase structure changes to a mixture of S- and R- phases.

Quantitative measurements of piezoelectric properties using IDS technology revealed piezoelectric coefficients of 29.3, 30, and 18.73 pm/V for the three samples, respectively. These results demonstrate a clear improvement in the piezoelectric performance of the BFO multilayer system, achieving values 2.79 to 4.47 times higher than that of the single-layer R-phase BFO film (6.7 pm/V). To explain the origin of the high piezoelectric response in the BFO multilayer system, we propose the following mechanism: The presence of a monoclinic phase with moderate tetragonality and the tunability of polarization rotation within this phase are critical factors (*16*, *35*). In particular, the intermediate bridging S-phase, with adjustable tetragonality in the range of 1.20 to 1.08, plays a critical role in enhancing the piezoelectric response. This phase not only exhibits suitable tetragonality but also facilitates easier polarization rotation, thereby substantially improving piezoelectric performance. On the other hand, the theoretical calculations combined with the phase-field simulation show that the increase of domain wall density also contributes to the improvement of piezoelectric performance. This high piezoelectric response mechanism could be analogously compared to the origins of high piezoelectricity in relaxor single crystal ferroelectrics (*36*).

By integrating experimental and theoretical approaches, this study provides profound insights into the structural evolution of BFO multilayer films under extreme compressive strain and the physical mechanisms underlying their enhanced piezoelectric properties. These findings offer valuable theoretical guidance for the design and optimization of high-performance lead-free piezoelectric materials.

In summary, we have revealed the ability to achieve high-performance piezoelectric by fabricating BFO/CCMO multilayer grown on LAO substrate, which show a different phase evolution path with single layer BFO grown on LAO substrate. Combined with atomic-scale studies and phase simulation studies, we have found that the phase/domain structure of BFO evolves sequentially from a periodic R/T mixed phase to a T/S mixed phase and lastly to an S/R mixed phase, as the thickness of BFO gradually decreases. By using IDS-PFM and phase-field simulation, it was found that the 16 U.C. sample showed excellent piezoelectric properties due to the T/S mixed phase, and its piezoelectric coefficient was substantially higher than that of the single-layer R-phase BFO, which was about four times higher. On the basis of comprehensive experimental results and phase-field simulations, we suggested that the high piezoelectric response in the BFO multilayer film system could be attributed to the moderate tetragonality of the S-phase, ease of polarization rotation, and the increase in domain wall density, providing theoretical support for the design of high-performance lead-free piezoelectric materials.

## MATERIALS AND METHODS

### Film growth

The LAO/(BFO/CCMO)_4_ films were deposited using a ComPexPRO 201FKrF (λ = 248 nm) excimer laser. Sintered BFO ceramic (~3 mol % Bi enriched) and CCMO ceramic targets were used for the (BFO/CCMO)_4_ multilayer film deposition. Before deposition, all substrates were preheated at 800°C for 10 min to clean the substrate surfaces and then cooled down to the growth temperature at a cooling rate of 15°C/min. A 10-min presputtering was used to clean the target surfaces. During the process of growing BFO film, the growth temperature was kept at 780°C, with a laser energy of 370 mJ, a laser frequency of 6 Hz, and under an oxygen pressure of 90 mtorr. For the growth of CCMO film, the growth temperature was kept at 780°C, with a laser energy of 270 mJ, a laser frequency of 4 Hz, and under an oxygen pressure of 75 mtorr. After depositing the films, the multilayer films were annealed at 780°C in an oxygen pressure of 200 torr for 5 min and then cooled down to RT at a cooling rate of about 5°C/min.

### TEM characterization

Cross-sectional samples for TEM experiments were prepared by slicing, gluing, grinding, dimpling, and, lastly, ion milling. A Gatan PIPS 691 was used for ion milling. The final ion milling voltage was less than 1 kV to decrease the amorphous layer, and the final milling angle was 5°. The dark-field images were acquired by FEI Tecnai F20. HAADF-STEM images were acquired using a Spectra 300 (FEI) microscope with a high-bright field emission gun and Cs corrector operating at 300 kV. The beam convergence angle was 25 mrad, and the collection angle ranges from 50 to 250 mrad. Strain analyses were based on GPA using Gatan Digital Micrograph software.

### Peak finding

The polarization direction of each BFO unit could be deduced by calculating the displacement vector of Fe (δ_Fe_) relative to the center of mass of the four nearest Bi neighbors, and the locations of Fe columns and Bi columns could be confirmed by fitting them as 2D Gaussian peaks based on the HAADF-STEM images; note here that the δ_Fe_ vector in each BFO unit cell is opposite to the corresponding polarization direction. All atomic-scale HAADF-STEM images have been drifted, corrected, and filtered.

### IDS-PFM and XRD measurements

IDS-PFM measurements were performed on a commercial PFM (Cypher, Asylum Research, USA) with an integrated quantitative laser doppler vibrometer system (Poly GmbH, Waldbronn, Germany) in ambient conditions at RT. Conductive diamond–coated silicon cantilevers (FM-LC) were used for IDS-PFM measurements. Since the operating voltage could be automatically divided by the operating voltage using programming statements during operation, the out-of-plane amplitude value is the out-of-plane piezoelectric coefficient *d*_33._

### XRD characterization

The phase purity and the crystallographic analysis of the thin films, that is, high-resolution XRD 2θ-ω and RSM, were carried out using a Panalytical X’Pert3 MRD diffractometer with Cu Kα_1_ radiation equipped with a 3D pixel detector.

### Phase-field modeling

Phase-field simulations were used to investigate the thickness-dependent evolution of domain structures in BFO film grown on the LAO substrate. The spatial distribution of the local polarization vector P*_i_* (*i* = 1, 2, and 3) serves as the order parameters, and its time-dependent evolution is governed by the following Ginzburg-Landau equation (*37*)

$$\frac{\partial P_{i}}{\partial t}=-L\frac{\delta F}{\delta P_{i}}$$

where *t* is the evolution time step, and *L* is the kinetic coefficient related to the mobility of the ferroelectric domain wall. The total free energy *F* of the system can be formulated as

$$F=∭\left(f_{\text{bulk}}+f_{\text{grad}}+f_{\text{elas}}+f_{\text{elec}}\right)\mathrm{dV}$$

where *f*_bulk_, *f*_grad_, *f*_elas_, and *f*_elec_ represent the bulk, gradient, elastic, and electric energy densities, respectively. Detailed expressions for these energy densities could be found in previous works (*37*, *38*). To describe the monoclinic Mc phase of BFO on the LAO substrate, an eighth-order Landau coefficient expansion is used, with coefficients sourced from prior studies (*39*, *40*) and listed in Table 1.

**Table 1.** Material parameters of BFO in the phase-field simulation.

Coefficient	Values	Coefficient	Values
$\alpha_{1}\left(N\;m^{2}\;C^{-2}\right)$	$-3.445\times 10^{8}$	$C_{11}\left(N\;m^{-2}\right)$	$2.28\times 10^{11}$
$\alpha_{11}\left(N\;m^{6}C^{-4}\right)$	$2.127\times 10^{9}$	$C_{12}\left(N\;m^{-2}\right)$	$1.28\times 10^{11}$
$\alpha_{12}\left(N\;m^{6}\;C^{-4}\right)$	$-2.049\times 10^{9}$	$C_{44}\left(N\;m^{-2}\right)$	$6.5\times 10^{10}$
$\alpha_{111}\left(N\;m^{10}\;C^{-6}\right)$	$-1.760\times 10^{9}$	$Q_{11}\left(m^{4}\;C^{-2}\right)$	0.071
$\alpha_{112}\left(N\;m^{10}\;C^{-6}\right)$	$8.298\times 10^{8}$	$Q_{12}\left(m^{4}\;C^{-2}\right)$	−0.03
$\alpha_{123}\left(N\;m^{10}\;C^{-6}\right)$	$1.679\times 10^{9}$	$Q_{44}\left(m^{4}\;C{-}^{2}\right)$	0.02015
$\alpha_{1111}\left(N\;m^{10}\;C^{-6}\right)$	$3.92\times 10^{8}$	$G_{11}/G_{110}$	0.6
$\alpha_{1112}\left(N\;m^{10}\;C^{-6}\right)$	$4.4\times 10^{7}$	$G_{12}/G_{110}$	−0.6
$\alpha_{1122}\left(N\;m^{10}\;C^{-6}\right)$	$-3.8\times 10^{8}$	$G_{44}/G_{110}$	0.6
$\alpha_{1123}\left(N\;m^{10}\;C^{-6}\right)$	$8.0\times 10^{8}$	$k_{b}$	45

The simulations are conducted on a discrete 3D mesh with dimensions of 128Δ*x* by 128Δ*y* by *n*Δ*z*, where the real grid spacing is set at Δ*x* = Δ*y* = 1 nm and Δ*z* = 0.2 nm. Here, *n* denotes the total number of grid points in the out-of-plane direction. The substrate and air layers are modeled with fixed thickness of 50Δz and 6Δz, respectively, while the thickness of the BFO film in the central region is varied from 2 to 9 nm. A thin-film mechanical boundary condition is applied, wherein the bottom film interface is coherently clamped by the substrate, and the top surface is assumed to be traction free (*37*). The in-plane directions use periodic boundary conditions, corresponding to the standard assumption of a laterally infinite epitaxial film in phase-field modeling. Along the out-of-plane direction, a thin-film mechanical constraint is adopted: The bottom interface of the BFO layer is coherently clamped to the substrate with fixed in-plane displacement, and the top surface is traction free. For the electrical boundary conditions, short circuit conditions are applied at both the top and bottom surfaces of the film.

The simulation begins with a downward polarization state, to which random noise with a small magnitude (<0.01 C/m^2^) is added to serve as nuclei for domain formation. To evaluate the effective small-signal piezoelectric response, we apply a series of weak out-of-plane electric fields (10, 30, 50, 70, and 90 kV/cm) that remain well below the coercive strength, thereby preserving the domain configuration and ensuring a linear electromechanical response. For each applied field, the resulting change in the averaged out-of-plane strain Δε_33_ is recorded, and the slope of Δε_33_ with respect to the applied field yields the effective small-signal piezoelectric coefficient (*41*): $d_{33}={\partial \Delta \varepsilon }_{33}/\partial E$, as shown in fig. S9.

### First-principles calculations

Density functional theory within the local spin-density approximation (LSDA) (*42*) was used, using the projector augmented-wave method (*43*) as implemented in the Vienna Ab initio Simulation Package (*44*). To correct the metallic behavior observed in the LSDA band structure, a Hubbard *U* parameter of 4 eV was applied to the Fe 3d states (*42*, *45*). To correct the metallic behavior observed in the LDA band structure, we have applied the Hubbard parameter *U* = 4 eV in our calculations. Fe’s 3p^6^3d^6^4s^2^, Bi’s 5d^10^6s^2^6p^3^, and O’s 2s^2^2p^4^ electrons were treated as valence electrons explicitly. We considered a 2 × 2 × 2 supercell containing 40 atoms that can accommodate all the possible antiferrodistortive rotations of the FeO_6_ octahedra. A plane-wave energy cutoff of 550 eV and a 4 × 4 × 4 Monkhorst-Pack k-point mesh were used in all calculations. Structural optimizations were achieved by allowing the atoms in the unit cell to relax until all the forces on each atomic site were below 0.002 eV/Å and simultaneously achieving a total energy convergence of 10^−6^ eV.
