# Supplementary material for: Thickness-confined metastable phase transitions drive large piezoelectricity in ultrathin BiFeO3
Source: Sci Adv. 2026 Mar 13;12(11):eaeb7174. doi: 10.1126/sciadv.aeb7174 (PMC12985725; doi:10.1126/sciadv.aeb7174)
Supplement: Supplementary file 1 — Figs. S1 to S9 Supplementary Text [file sciadv.aeb7174_sm.pdf]

Science Advances
AAAS

Supplementary Materials for

Thickness-confined metastable phase transitions drive large piezoelectricity in ultrathin $BiFeO_3$

Shuang-Jie Chen *et al.*

Corresponding author: Yunlong Tang, yltang@imr.ac.cn

Sci. Adv. **12**, eaeb7174 (2026)
DOI: 10.1126/sciadv.aeb7174

This PDF file includes:

Figs. S1 to S9
Supplementary Text

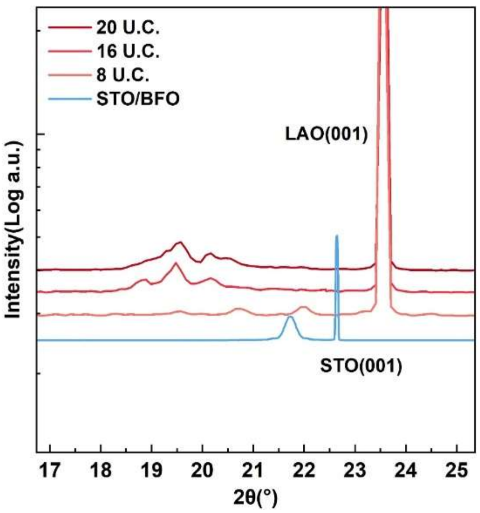

Figure S1. XRD θ-2θ scans of the LAO/(BFO/CCMO)$_4$ films with different thickness and STO/BFO films.

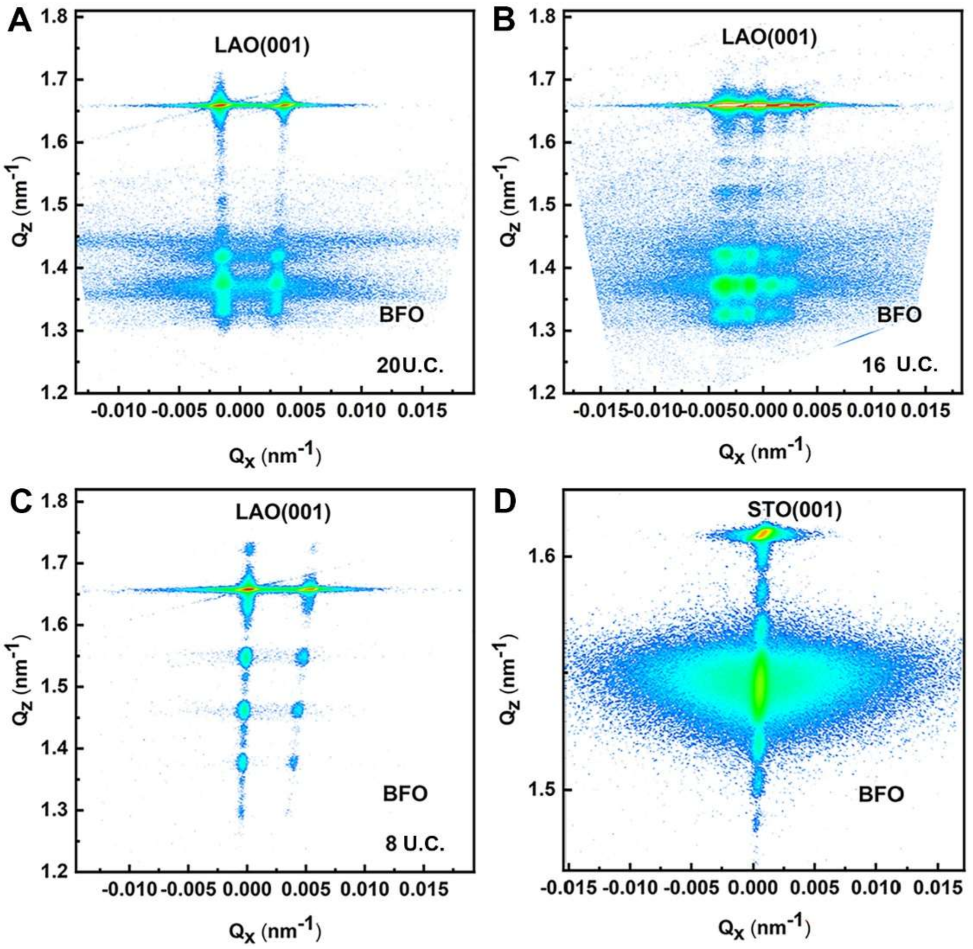

Figure S2. (A-D) RSM around the (001) substrate reflection of LAO/(BFO/CCMO)$_4$ films with different thicknesses (20 U.C., 16U.C., 8 U.C.) and STO/BFO films, respectively. BFO could form a tetragonal-like phase with a large axial ratio under large compressive strain, based on **Fig. S1** and **S2**.

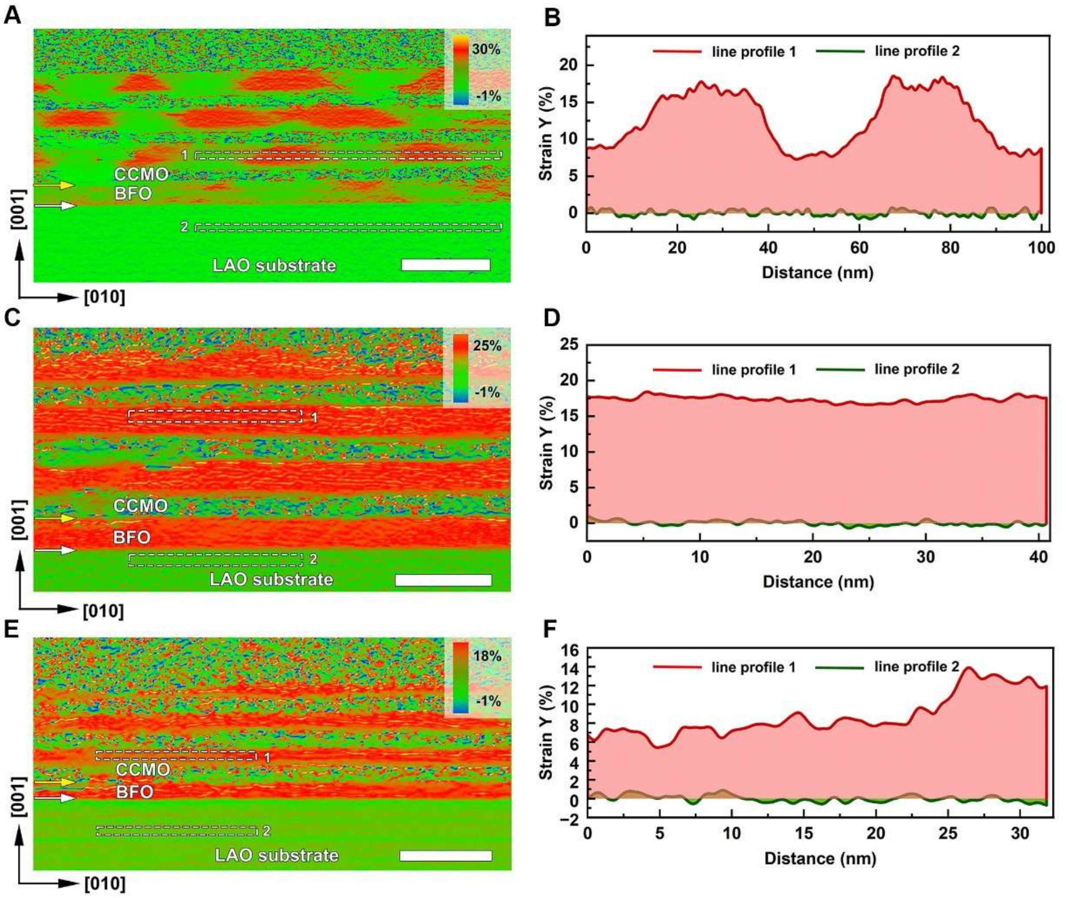

Figure S3. Out-of-plane strain analysis of LAO/(BFO/CCMO)$_4$ films with different thicknesses corresponding to Fig. 1(A, C, E) via GPA and their quantitative analysis. (A-B) Out-of-plane strain of **Fig. 1A** and its corresponding quantitative analysis. **(C-D)** Out-of-plane strain of **Fig. 1C** and its corresponding quantitative analysis. **(E-F)** Out-of-plane strain of **Fig. 1E** and its corresponding quantitative analysis. Scale bars, 30 nm (**A**); 20 nm (**C** and **E**).

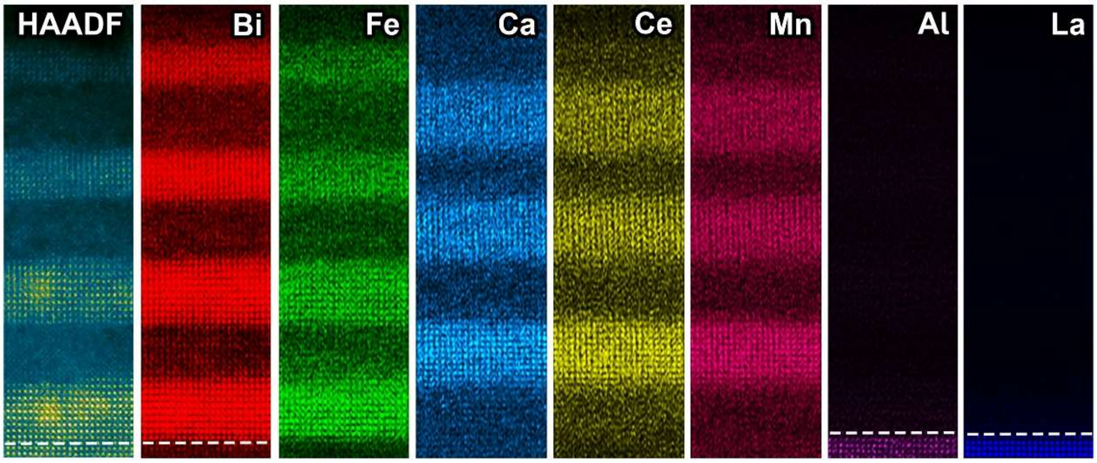

Figure S4. Elemental composition maps of Bi, Fe, Ca, Ce, Mn, Al and La in LAO/(BFO/CCMO)$_4$ with 8 U.C. monolayer BFO.

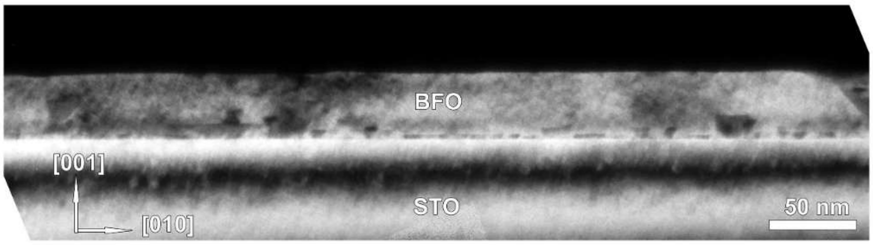

Figure S5. A dark-field TEM image of STO/BFO with a thickness of 40 nm.

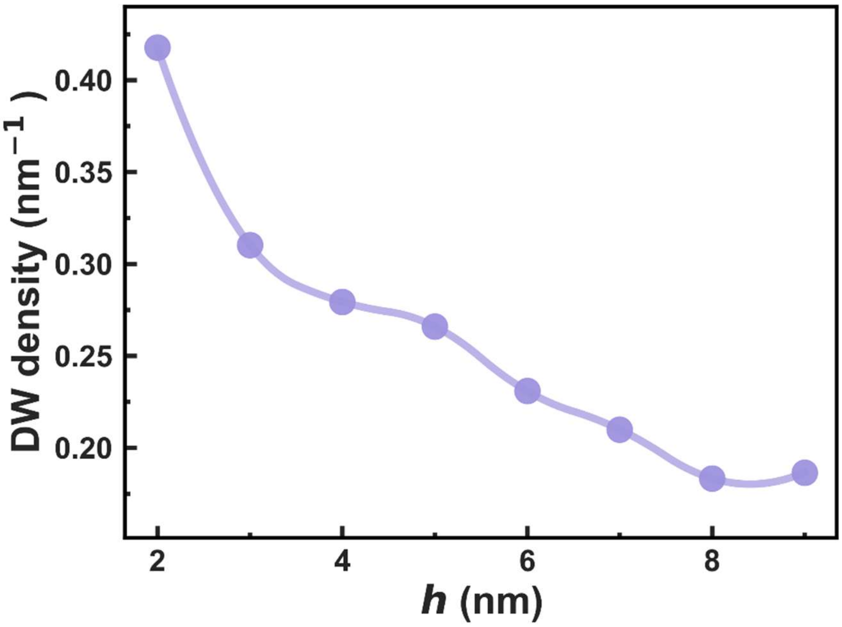

Figure S6. Evolution of domain density as a function of film thickness from phase field simulation.

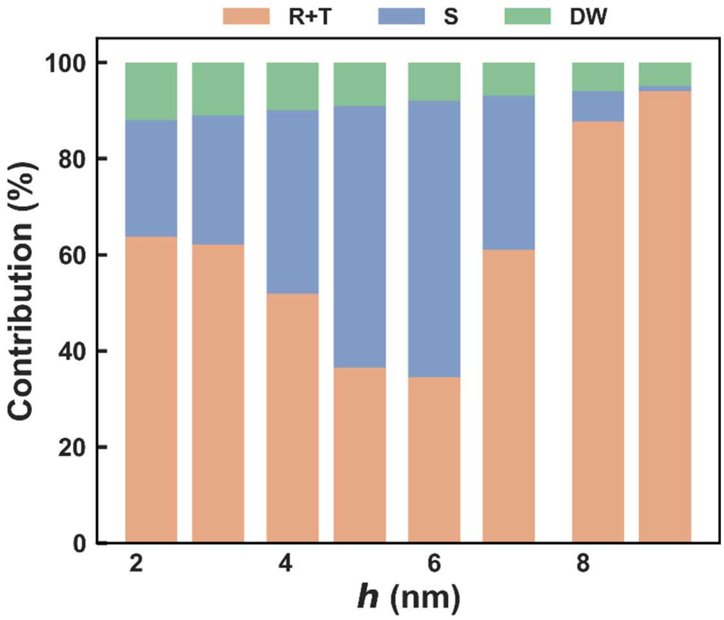

Figure S7. Relative contribution fractions of the S phase, the R+T phases, and the DW component at each film thickness.

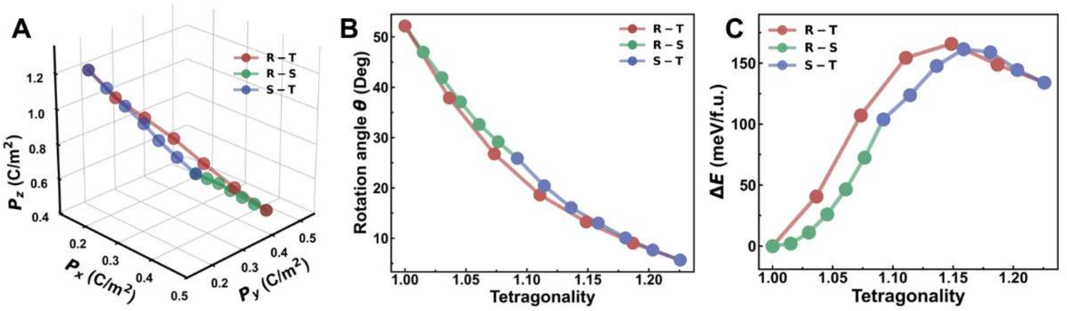

Figure S8. SSNEB analysis of polarization rotation and energy barriers near the MPB in BFO. **(A)** SSNEB-calculated polarization paths between R-T, R-S, and T-S phases in polarization space (P_x, P_y, P_z), showing smooth transition trajectories. **(B)** Rotation angle θ, defined as the angle between the polarization vector and the [001] axis, plotted versus tetragonality. **(C)** Relative energy difference ΔE along each path versus tetragonality, where ΔE is calculated with respect to the R phase.

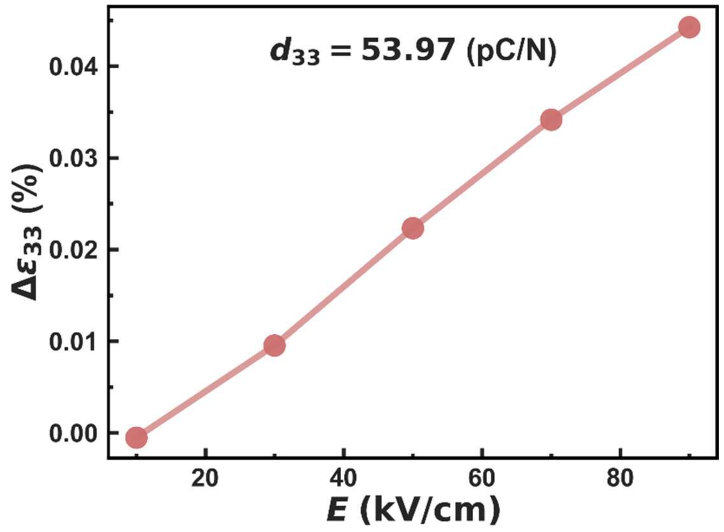

Figure S9. Determination of the effective piezoelectric coefficient from phase-field simulation via linear response analysis. The field-induced change in the average out-of-plane strain component ε_{33} is plotted as a function of the applied electric field. The slope obtained from linear fitting corresponds to the effective piezoelectric coefficient: $d_{33} = \partial\Delta\varepsilon_{33}/\,\partial E$. The result shown corresponds to a BFO film thickness of 5 nm.

Supplementary Text

The contribution of each structural component to the piezoelectric response is then evaluated by weighing the calculated piezoelectric response with the corresponding phase fraction and averaging over the in-plane area, as defined by:

$$d_{33}^{(\alpha)} = \frac{1}{A}\int f_\alpha\,(x,y)\; d_{33}(x,y)\; dxdy$$

Here, A is the area of the xy plane, $f_\alpha(x, y)$ is the local volume fraction of each component α (α = R, T, S, or DW), and $d_{33}(x,y)$ is the local piezoelectric coefficient.
